# Structure and Diversity of Soil Bacterial Communities in Offshore Islands

**DOI:** 10.1038/s41598-019-41170-9

**Published:** 2019-03-20

**Authors:** Yu-Te Lin, Yu-Fei Lin, Isheng J. Tsai, Ed-Haun Chang, Shih-Hao Jien, Yen-Ju Lin, Chih-Yu Chiu

**Affiliations:** 1 Biodiversity Research Center, Academia Sinica, Nankang, Taipei 11529 Taiwan; 2 Mackay Junior College of Medicine, Nursing and Management, Beitou, Taipei 11260 Taiwan; 3 Department of Soil and Water Conservation, National Pingtung University of Science and Technology, Pingtung, 91201 Taiwan

## Abstract

The effects of biogeographical separation and parent material differences in soil bacterial structure and diversity in offshore islands remain poorly understood. In the current study, we used next-generation sequencing to characterize the differences in soil bacterial communities in five offshore subtropical granite islands (Matsu Islets, MI) of mainland China and two offshore tropical andesite islands (Orchid [OI] and Green Islands [GI]) of Taiwan. The soils of OI and GI were more acidic and had higher organic carbon and total nitrogen content than MI soils. The bacterial communities were dominated by *Acidobacteria* and *Proteobacteria* but had different relative abundance because soils were derived from different parent material and because of geographic distance. Non-metric multi-dimensional scaling revealed that the communities formed different clusters among different parent material and geographically distributed soils. The alpha-diversity in bacterial communities was higher in tropical than subtropical soils. Mantel test and redundancy analysis indicated that bacterial diversity and compositions of OI and GI soils, respectively, were positively correlated with soil pH, organic carbon, total nitrogen, microbial biomass carbon and nitrogen. These results suggest that variations in soil properties of offshore islands could result from differences in soil parent material. Distinct soils derived from different parent material and geographic distance could in turn alter the bacterial communities.

## Introduction

Soil bacteria are essential parts of soil ecosystems and are involved in mineralizing organic matter, biogeochemical cycling of carbon and nitrogen and many other soil processes^1–3^. Their distribution can be altered by soil properties^4,5^, plant species^6^, litter quality and root exudates^7–9^ as well as temperature and precipitation in different climatic conditions^10,11^. For instance, bacterial phylotype richness and composition, including the relative abundance of *Acidobacteria*, *Actinobacteria*, and *Bacteroidetes*, is significantly correlated with soil pH^4^. Bacterial communities cluster separately among four different tropical tree species^6^. Recently, studies of microbial biogeography have focused on the microbial communities across spatial distance^12,13^. Other than revealing a causal relation between microbes and their environments, such studies could also provide insights into possible environmental drivers of change in microbial communities^14^.

Our previous study using clone libraries revealed similar soil bacterial communities in two isolated islands, but climate conditions and soil characteristics affected the bacterial community composition between offshore islands and inland soils of Taiwan^15^. However, this approach with a low number of sequences provided little resolution for estimating diversity and structure of bacterial communities. The work could also be site-specific, providing less information about bacterial communities across biomes and regions.

The current study investigated the island soil bacterial diversity and structure by biogeographical separation and parent material differences based on high-throughput sequencing. The offshore, subtropical granite islands of China, Matsu Islets (MI), were under long-term military control and restricted industrial activities. Some ecological studies were conducted in this region^16–18^; however, no study has focused on the soil microbial diversity in these islets. We included two more tropical andesite islands of Taiwan, Orchid Island (OI) and Green Island (GI), for comparison. For more detailed and comprehensive information on soil bacterial biogeography, we examined such broad and diverse regions across larger spatial and climatic scales. Our first objective was to elucidate the bacterial diversity and structure of these offshore islands with different soil properties. The second objective was to compare the structure of bacterial communities in soils derived from different parent material and with geographical isolation among these islands. This research could provide important information for understanding how soil bacterial communities respond to environmental factors on these islands.

## Results

### Soil properties

Basic properties of soil samples from the surveyed islands are listed in Table 1. MI soils were more acidic and had less organic carbon (org. C) and total nitrogen (TN) content than other soils. Both microbial biomass C (MBC) and N (MBN) were significantly reduced in MI soils (*P* < 0.05). OI and GI are characterized by higher pH, org. C, TN, MBC and MBN as compared with MI soils. Microbial biomass phosphorus (MBP) content was significantly higher in GI soils than other island soils (*P* < 0.05) (Table 1). Soils in MI were classified as Typic Haplustult according to the US Soil Taxonomy^19^, whereas soils on GI and OI are Typic Paleudult, which reflects a relative discrepancy in climate between MI and GI or OI.

**Table 1:** The soil properties of Matsu Islets, Orchid Island and Green Island^a^.

Island	Site	pH	Org. C	TN	MBC	MBN	MBP	Soil texture	Soil type
			(g kg^−1^)	(g kg^−1^)	(μg C g^−1^ S)	(μg N g^−1^ S)	(μg N g^−1^ S)		
Matsu	BG	4.2 ± 0.3c	57.1 ± 12.3c	4.8 ± 1.0b	641.7 ± 54.8c	91.9 ± 14.7 cd	26.9 ± 2.5b	Clay loam	Haplustult
	NG	4.9 ± 0.3b	24.0 ± 3.8e	1.7 ± 0.2e	448.5 ± 59.5de	71.3 ± 10.3de	10.5 ± 2.1d	Sandy clay loam	Haplustult
	DJ	4.4 ± 0.1c	23.8 ± 3.0e	1.5 ± 1.0e	412.7 ± 61.0e	63.5 ± 9.4e	20.3 ± 5.0b	Sandy loam	Haplustult
	SJ	4.8 ± 0.4bc	23.0 ± 2.8e	2.0 ± 0.1d	503.9 ± 22.2d	81.4 ± 6.7d	22.8 ± 10.1bc	Clay loam	Haplustult
	DY	4.8 ± 0.2bc	29.0 ± 1.4d	2.5 ± 0.1c	565.8 ± 26.7c	95.5 ± 4.2c	13.7 ± 0.8c	Silty clay loam	Haplustult
Orchid	OI	6.1 ± 0.5a	64.0 ± 7.6b	5.1 ± 0.9ab	1241.6 ± 588.6b	234.2 ± 113.7b	12.1 ± 7.1 cd	Silty clay	Paleudult
Green	GI	6.0 ± 0.0a	76.0 ± 2.3a	6.2 ± 0.3a	1963.5 ± 326.5a	444.3 ± 39.2a	63.9 ± 15.3a	Sandy clay loam	Paleudult

^a^The data shown are the mean ± standard deviation of four replicates (eight replicates for OI). Data with the different letter in each column indicates significant differences among sites according to LSD-test at *p* < 0.05. Sites: BG, Beigan; NG, Nangan; DJ, Dongju; SJ, Shiju; DY, Dongyin; OI, Orchid Island; GI, Green Island.

### Bacterial community composition

**Figure 1:**
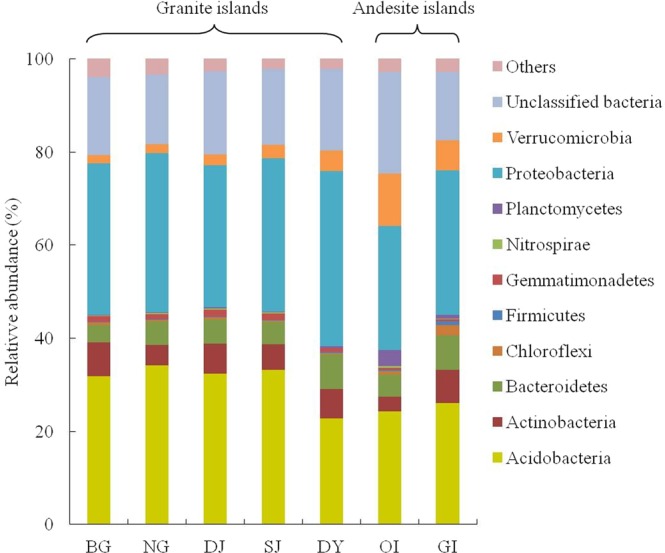
Relative abundance of bacterial phyla among soil bacterial communities for all offshore islands. Site abbreviations are in Table 1.

The major phyla obtained from Miseq libraries (a total of 84,000 sequences for GI and each island of MI; 168,000 sequences for OI) were *Acidobacteria* (24–34%) and *Proteobacteria* (27–38%) (Fig. 1). *Actinobacteria* was the third most abundant phylum and comprised only 3% to 7% of the communities. Other phyla, such as *Bacteroidetes*, *Gemmatimonadetes* and *Verrucomicrobia*, each accounted for less than 8% of the communities (Fig. 1). Among *Acidobacteria*, groups (Gp) 6, 1, and 2 were the most abundant, and the relative abundance of Gp6 was greater than 6%, except in Dongyin (DY) communities (Table 2). Within *Proteobacteria*, *Alphaproteobacteria* was the most abundant class (18–39%), followed by *Gammaproteobacteria* and *Betaproteobacteria*. Among communities, *Phenylobacterium* (1.1%) of *Caulobacterales* and *Bradyrhizobium* (0.5%) of *Rhizobiales* were the two most abundant genera of *Alphaproteobacteria*. *Burkholderia* (0.9%) was the most abundant genus of *Betaproteobacteria*, and its relative abundance was higher in granite than andesite island communities (Table 2).

**Table 2:** The relative abundance (%) of some abundant genera and genus-level groups of offshore island soil bacterial communities. Site abbreviations are in Table 1.

Affiliation	BG	NG	DJ	SJ	DY	OI	GI	% of all reads
*Acidobacteria* Gp6	7.0	6.1	7.8	9.7	4.4	8.5	10.0	7.6
*Acidobacteria* Gp1	3.9	7.3	5.4	3.9	3.5	2.0	2.1	4.0
*Acidobacteria* Gp2	4.5	4.6	3.6	3.9	5.0	2.3	1.5	3.6
Saccharibacteria_genera_incertae_sedis	4.5	3.4	2.9	2.3	2.7	0.0	0.9	2.4
WPS-2_genera_incertae_sedis	3.9	2.1	2.9	2.2	5.3	0.0	0.3	2.4
*Verrucomicrobia* Subdivision3_genera_incertae_sedis	1.4	1.0	1.6	2.2	3.0	4.8	2.0	2.3
*Acidobacteria* Gp3	1.8	2.8	2.3	2.3	2.0	0.5	1.2	1.8
Spartobacteria_genera_incertae_sedis	0.6	1.2	1.0	1.1	1.3	4.1	3.6	1.8
*Gemmatimonas*	2.0	1.8	2.2	2.3	1.4	0.3	0.4	1.5
*Acidobacteria* Gp4	0.7	0.6	1.6	1.1	0.1	2.2	3.5	1.4
*Phenylobacterium*	1.3	1.4	0.9	1.4	2.1	0.2	0.3	1.1
*Gaiella*	0.8	0.5	0.8	0.8	1.4	0.7	1.5	0.9
*Acidobacteria* Gp7	0.3	0.9	0.9	0.9	0.5	1.8	0.8	0.9
*Burkholderia*	1.2	1.6	0.9	1.0	1.2	0.1	0.2	0.9
*Acidobacteria* Gp5	0.1	0.3	0.4	0.3	0.2	1.6	0.9	0.5
*Acidobacteria* Gp13	1.4	0.2	0.4	0.7	0.5	0.2	0.2	0.5
*Bradyrhizobium*	0.4	0.5	0.4	0.4	0.3	0.3	0.8	0.5

The relative abundance among communities showed some variation, despite a similar abundant phyla. Within MI communities, *Acidobacteria* comprised more than 30% of sequences, except in DY soils. However, the proportion of *Acidobacteria* in OI and GI soils was only about 25%. The relative abundance of *Proteobacteria* revealed a similar trend — more than 32% of MI communities, and only about 26% to 30% of OI and GI communities. Within *Proteobacteria*, the relative abundance of *Alphaproteobacteria* and *Gamaproteobacteria* was higher in MI than OI and GI soil communities. The phylum *Verrucomicrobia* accounted for 6% to 11% of OI and GI communities but less than 5% of all MI soils.

**Figure 2:**
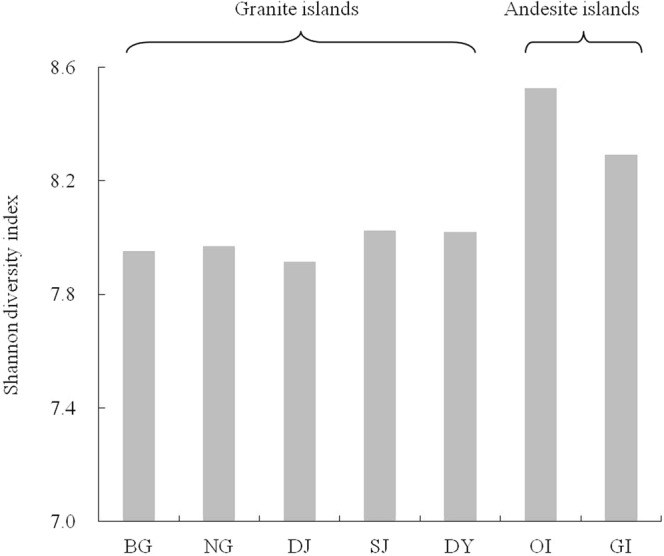
Shannon diversity index of soil bacterial communities for all offshore islands. Operational taxonomic units (OTUs) were calculated at the 3% evolutionary distance. Site abbreviations are in Table 1.

*K*-shuff analysis of the soil bacterial communities also revealed differences among these offshore islands (Table S1). The non-metric multidimensional scaling (NMDS) plot derived from *C*_kf_ of *K*-shuff analysis indicated that the community structures among MIs did not significantly differ from each other but significantly differed from OI and GI structures (Fig. 2, Table S1).

### Bacterial community diversity

**Figure 3:**
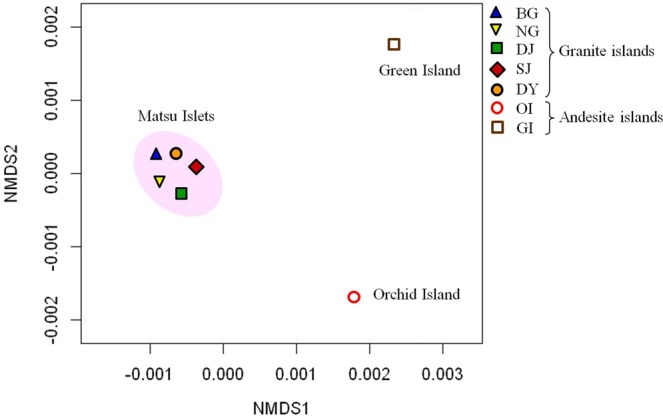
Non-metric multidimensional scaling plot derived from *C*_kf_ of *K*-shuff analysis. Site abbreviations are in Table 1.

**Figure 4:**
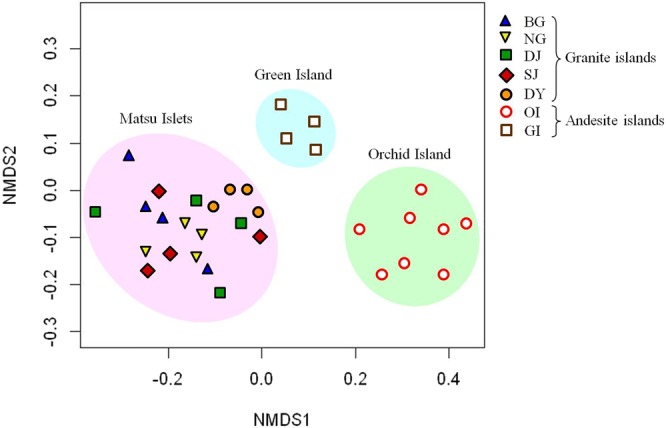
Non-metric multidimensional scaling plot of soil communities for all offshore islands. Site abbreviations are in Table 1.

Besides the distinctive community composition, the bacterial alpha-diversity among these communities (a total of 84,000 sequences for GI and each island of MI; 168,000 sequences for OI) also differed. Calculated at an evolutionary distance <0.03 (about 97% sequence similarity), the Shannon diversity index revealed that the bacterial diversity was similar among the individual islands of MI (Fig. 3). The OI community was more diverse than the GI community and the diversity was higher for OI and GI than MI communities (Fig. 3). The results of Chao 1 estimator and rarefaction curve analyses supported these results (Fig. S1). β-diversity, analyzed by community comparison of the NMDS plot indicated the different clusters formed between subtropical granite and tropical andesite islands (Fig. 4). NMDS plot analysis also revealed differences between OI and GI communities, thereby suggesting that the different soil properties affect bacterial communities on these two andesite islands. Taken together, MI communities showed close distribution, whereas OI and GI communities formed two separate clusters. These results reflect the close geographic distribution and soil parent material of MI and the distant geographic isolation and soils derived from different soil parent materials among MI, OI and GI soils.

### Relation between bacterial communities and soil properties

**Figure 5:**
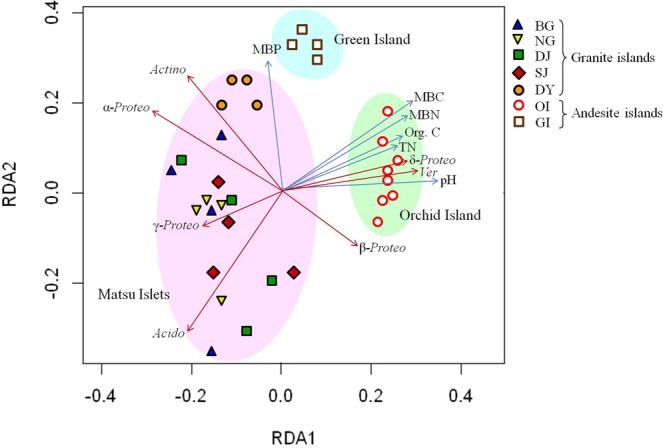
Redundancy analysis of bacterial communities in all offshore islands. Site abbreviations are in Table 1. Abbreviations in figure: Org. C, organic carbon; TN, total nitrogen; MBC, microbial biomass carbon; MBN, microbial biomass nitrogen; MBP, microbial biomass phosphorus; *Acido*: *Acidobacteria*; *Actino*, *Actinobacteria*; α-*Proteo*, *Alphaproteobacteria*; β-*Proteo*, *Betaproteobacteria*; γ-*Proteo*, *Gammaproteobacteria*; δ-*Proteo*, *Deltaproteobacteria*; *Ver, Verrucomicrobia*.

The community composition of MI soils was significantly altered by the soil properties of TN, MBC and MBN. In contrast, only soil pH was correlated with bacterial diversity (Table S2). Similarly, soil org. C, MBC, MBN and MBP were significantly correlated with OI and GI community composition, but their bacterial diversity was correlated with all soil properties, except for soil acidity (Table S2). Soil pH, org. C, TN, MBC, MBN and MBP significantly altered the composition and alpha-diversity of bacterial communities on the Mantel test (Table S2). Redundancy analysis (RDA) was further used to elucidate the relation between bacterial composition and soil properties. The bacterial composition was positively associated with soil properties for OI and GI soils (Fig. 5). The distribution of some of the most abundant taxa, *Acidobacteria*, *Actinobacteria*, *Alphaproteobacteria* and *Gammapreoteobacteria*, was negatively correlated with these soil characteristics, except MBP. The distribution of other phylogenetic groups, including *Betaproteobacteria*, *Deltaproteobacteria* and *Verrucomicrobia*, was positively correlated with soil pH, org. C, TN, MBC and MBN. In addition, on combining the replicates of each site for RDA, the results further confirmed the different clusters between MI, OI and GI soils and the correlations between communities and soil properties (Fig. S2).

## Discussion

The soil bacterial communities of offshore, isolated islands examined in this study were all dominated by *Acidobacteria* and *Proteobacteria*, but the relative abundance differed. The bacterial diversity also varied among MI, OI and GI soil communities. OI and GI have higher annual temperature and precipitation than MIs. Higher temperature and precipitation could shape the bacterial communities^20^ and result in greater bacterial diversity in tropical soils. In addition, higher org. C and total nitrogen content in tropical island soils could provide sufficient nutrient availability to affect bacterial community composition^21^.

The soil parent material could be another key factor affecting soil bacterial communities among MI, OI and GI soils. Soil parent material could provide a basic nutritional environment for the development of microbial communities^22^. One study showed that soil bacterial structure is determined by the nature of the parent material, and the community still maintained characteristics of the original populations even after a long time^23^. The soil mineralogy of soils derived from different parent materials, granite and andesite, is distinct and strongly controls the soil C processes in temperate forest soils^24^. It also affects the accumulation of soil microbially derived residues^25^, which suggests that soil microbial communities differ under different parent materials. A study found variable reactions of different bacterial phylotypes, and operational taxonomic units (OTUs) affiliated with *Acidobacteria*, *Actinobacteria*, and *Alpha*- and *Betaproteobacteria* mainly responded to granite soils^26^. Moreover, the soils had more neutral pH values on OI and GI than MI. The increased abundance of *Acidobacteria* in MI soils and the negative correlation between *Acidobacteria* distribution and soil pH based on RDA agree with previous studies^27,28^. These results support the importance of soil pH affecting soil communities across spatial scales^5^ and in local regions. The soil community variability among these offshore islands could have resulted from the quite different soil parent materials and were determined from the early stage of soil formation. The non-random distribution in soil bacteria could reflect the correlation with key environmental variables across different spatial scales^29,30^.

The variation in bacterial communities among these island soils could also reveal the effect of geographic distance. The distance from MI to OI and GI is >400 km. Some studies have revealed a significant effect of distance at intermediate scales of 10 to 3,000 km on soil community composition^31,32^, and environmental differences also had effects on the communities at this scale^31^. In the present study, the geographic distances among island soils were at the intermediate scale, so both environmental differences and geographic distances could alter the soil communities. In addition, the same cluster formed by the communities of MI indicated that the local environmental properties could be more essential factors affecting bacterial communities.

*Acidobacteria* Gp6 was the most abundant genus-level group of *Acidobacteria*, followed by Gp1, 2, 3 and 4. The relative abundance of *Acidobacteria* Gp4 and 6 was higher in OI and GI communities, whereas that of *Acidobacteria* Gp1, 2, 3 was higher in MI soils. The abundance of *Acidobacteria* Gp1, 2 and 3 is negatively related to soil pH, and that of *Acidobacteria* Gp4 and 6 is positively related to soil pH^33^. The differences in abundance among these *Acidobacteria* subdivisions could reflect the responses of community composition to the soil properties. It also indicates the regulation of soil pH for this phylum and its individual subdivisions.

*Alphaproteobacteria* was the most abundant class of the *Proteobacteria* in these island soils, although relative abundances differed among communities. Many sequences are affiliated with the genus *Bradyrhizobium*, order *Rhizobiales*, which include species that could perform nitrogen fixation, organic matter decomposition, and plant growth promotion^34,35^. This group also includes a number of nonsymbiotic bacteria with varied biochemical functions such as denitrification^36^ and photosynthesis^37^. Their versatile metabolism ability suggests their importance in nutrient availability to the soil ecosystems, especially in GI communities. Among *Betaproteobacteria*, the genus *Burkholderia* was abundant and is found in rhizosphere soil communities that perform nitrogen fixation and plant growth promotion^38^. On the basis of their physiological characteristics, they could be involved in parts of the carbon and nitrogen biogeochemical cycles in these offshore island soil ecosystems. In addition, *Burkholderia* was more abundant in MI than OI and GI soil communities, perhaps because MI soils have low nitrogen content and the genus is able to fix nitrogen under these conditions.

The relative abundance of *Verrucomicrobia* in OI soils was especially high among these communities. This phylum has a wide distribution across a range of biomes^39^. A recent study indicated that their distribution was significantly affected by soil pH, total carbon and nitrogen, and carbon/nitrogen ratio^40^. The high soil pH and total carbon and nitrogen content in OI and GI soil may explain why *Verrucomicrobia* was abundant on these islands and not MI. Moreover, this phylum includes members involved in polysaccharide degradation^41^ and nitrogen fixation^42^, so they may have essential roles in the tropical soil ecosystem. It was also an abundant phylum in the soil community analyzed with the clone library method^15^. Members of this phylum could be further isolated to elucidate their roles in soils of these tropical islands.

The genus *Gemmatimonas* of *Gemmatimonadetes* was one of the abundant genera in offshore island soil communities. This group comprises about 2.2% of soil communities analyzed by high-throughput sequencing^43^. *Gemmatimonas* was relatively more abundant in MI than OI and GI soil communities. The finding could reflect the adaption to low soil moisture of this group^43^ because of the reduced annual precipitation in MI. Moreover, an obligate aerobic *Gemmatimonas* strain had a unique regulatory mechanism of N_2_O reduction^44^, which indicates the potential role of this genus in nitrogen cycling in this ecosystem, especially in MI soils.

In conclusion, bacterial diversity was higher in tropical andesite than subtropical granite island soil communities. The major phyla were similar, but their relative abundance among communities differed. The subtropical granite and tropical andesite island soil communities formed different clusters and had distinct relations with soil properties, including soil pH, org. C, TN, MBC, MBN, and MBP. These results suggest that different parent material and geographic distance affected soil properties and altered the bacterial composition and diversity among these offshore island soils. In the local region, the environmental properties could be the more essential factors affecting bacterial communities, whereas environmental differences and geographic distances could both alter the soil communities across a large scale.

## Materials and Methods

### Site description and soil sampling

**Figure 6:**
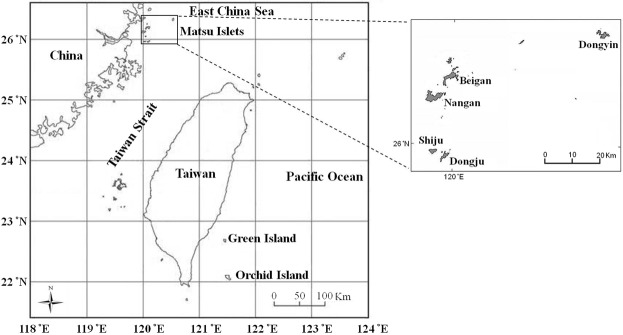
Sampling sites at offshore islands.

This study was conducted on offshore islands, including MI of subtropical granite and OI and GI of tropical andesite (Fig. 6). The map was prepared on the basis of State Information System database, Taiwan (https://ngis.wra.gov.tw/NgisGIS/). MIs are in the East China Sea and located 10–50 km off southeastern China, about 210 km northwest of Taiwan, facing the Taiwan Strait. Five islands in this region were used for sampling: Beigan (BG) (26°22′N, 119°99′E), Nangan (NG) (26°15′N, 119°93′E), Dongju (DJ) (25°95′N, 119°97′E), Shiju (SJ) (25°97′N, 119°94′E), and DY (26°36′N, 120°49′E) islands. BG and NG, DJ and SJ are close to one another; DY is more distant from other four islands. These islands have a subtropical marine climate with mean precipitation about 1,000 mm and annual average temperature 18.6 °C. The soil parent material is granite and the environments have remained little disturbed since broadleaved forests were established about 5 decades ago. OI (22°01′N, 121°34′E) is in the Pacific Ocean and located at about 60 km off the southeastern coast of Taiwan and facing the Pacific Ocean. It is a tropical island with mean precipitation >3000 mm and annual average temperature 22.6 °C. The vegetation is natural and consists of a little-disturbed secondary broadleaved forest. GI (22°39 N, 121°29) is also in the Pacific Ocean and located about 30 km off the eastern coast of Taiwan, facing the Pacific Ocean. A secondary broadleaved forest is the main vegetation. The mean temperature is 23.5 °C and mean precipitation about 2,500 mm. The lands chosen as sampling locations for this study are owned by the Forestry Bureau and accessible to the public. Research proposals were approved and granted by the Ministry of Science and Technology, Taiwan. Field and laboratory studies did not involve any animal husbandry, nor any protected or endangered biological species. The surface soil (0–10 cm) of each island was sampled in four replicates (eight replicates for OI because of a much larger area) of 50 × 50-m plots established 50-m apart along transect lines. After removing the surface litter, three subsamples at each plot were collected by using a soil auger, 8 cm in diameter and 10 cm deep, and pooled. The soil samples were sieved with a 2-mm sieve to remove the debris of litter and gravel and one part was kept at 4 °C before biochemical and molecular assays. Other samples were freeze-dried and stored at −20 °C for DNA extraction.

### Soil analysis

Soil pH was determined in a 1:1 soil/water slurry by using a portable Jenco 6009 pH/mV meter^45^. Soil org. C and TN were examined by the combustion method with an NCS elemental analyzer (Model NA1500 Fisons, Italy). Soil MBC and MBN were assayed by using the chloroform fumigation extraction method as described^46^. In brief, soil samples were equally separated into two sets. One set was fumigated with chloroform for 24 hr and extracted with 0.5 M potassium sulphate (K_2_SO_4_). The other set was directly extracted with 0.5 M K_2_SO_4_. MBC and MBN were calculated as the differences between the fumigated and unfumigated K_2_SO_4_-extractable C and N by multiplying coefficients (K_EC_ = 2.22 and K_EN_ = 4.95) [(fumigated C (N) − unfumigated C (N)) × coefficients]^47,48^.

### DNA extraction, PCR amplification, and high-throughput sequencing

Total soil DNA was extracted from 0.3 g soil samples with the PowerSoil DNA extraction kit (MoBio Laboratories, Carlsbad, CA) as directed by the manufacturer’s instructions. PCR amplification was performed according to the Illumina 16S Metagenomic Sequencing Library preparation guide (Illumina) with slight modification, targeting the V4 region (primer 515f and 806r) of the bacterial 16S rRNA gene with index sequences and adapters. PCR involved initial denaturation at 95 °C for 2 min, followed by 30 cycles of denaturation at 95 °C for 20 s, annealing at 55 °C for 15 s, elongation at 72 °C for 5 min, and a final elongation at 72 °C for 10 min. Paired-end sequencing of the PCR amplicons involved the MiSeq 250-bp paired sequencing system (Illumina) according to the manufacturer’s instructions.

### Sequence data analyses

Illumina sequencing data were pair-assembled using PEAR^49^. The data were then processed with the Ribosomal Data Project (RDP) pipeline (http://pyro.cme.msu.edu; RDP release 11.5; release date: 2016.09.30). The sequences that did not have Ns and were >200 bp long, were free of chimeras by using Uchime^50^, and had quality scores >25 were used for further analysis. Taxonomic information was analyzed by using the Naïve Bayesian rRNA classifier^51^ of the RDP pipeline with a confidence cutoff of 80%. Data at the genus level were also examined and the Miseq 250-bp paired sequencing system could be used to analyze communities at this level with high accuracy^52^. The RDP pipeline was also used to calculate the Shannon diversity index^53^ based on the Complete Linkage Clustering data for OTUs with an evolutionary distance of 0.03. To avoid measurement differences for estimating the alpha and beta diversity from sample size, the sequences for each replicate were normalized to 21,000 sequences with random selection from the larger samples for community comparisons. *K*-shuff analysis was used for community comparisons^54^. NMDS community comparisons and relations between bacterial composition, diversity and soil properties were assessed by using PRIMER v6^55^. RDA involved the vegan package in R v.3.2.1 to determine the relation between bacterial composition and soil properties. All sequences were deposited in MG-RAST (Metagenome Rapid Annotation using Subsystems Technology)^56^ with accession numbers 4812289.3, 4812290.3 and 4812291.3.

## Supplementary information

Table S1, S2, and Figure S1, S2
