# Supplementary material for: Structure and Diversity of Soil Bacterial Communities in Offshore Islands
Source: Sci Rep. 2019 Mar 20;9:4689. doi: 10.1038/s41598-019-41170-9 (PMC6426884; doi:10.1038/s41598-019-41170-9)

**Structure and Diversity of Soil Bacterial Communities in Offshore Islands**

**Yu-Te Lin^1^, Yu-Fei Lin^1^, Isheng J. Tsai^1^, Ed-Haun Chang^2^, Shih-Hao Jien^3^, Yen-Ju Lin^1^ and Chih-Yu Chiu^1^***

^1^ Biodiversity Research Center, Academia Sinica, Nankang, Taipei 11529, Taiwan

^2^ Mackay Junior College of Medicine, Nursing and Management, Beitou, Taipei 11260, Taiwan

^3^ Department of Soil and Water Conservation, National Pingtung University of Science and Technology, Pingtung 91201, Taiwan

*** Correspondence:** Chih-Yu Chiu, Biodiversity Research Center, Academia Sinica, Nankang, Taipei 11529, Taiwan. Tel.: +886 2 2787 1068; e-mail address: bochiu@sinica.edu.tw

**Table S1.** Statistical significance (*P*-value) of differences among offshore island soil communities by using *K*-shuff analysis^a^. Site abbreviations are described in Table 1.

|  | BG | NG | DJ | SJ | DY | OI | GI |
| --- | --- | --- | --- | --- | --- | --- | --- |
| BG | - | 0.711 | 0.307 | 0.245 | 0.238 | **0.002** | **0.016** |
| NG | 0.0014 | - | 0.444 | 0.353 | 0.154 | **0.001** | **0.02** |
| DJ | 0.0016 | 0.0012 | - | 0.696 | 0.418 | **0.001** | **0.004** |
| SJ | 0.0017 | 0.0018 | 0.0004 | - | 0.32 | **0.001** | **0.001** |
| DY | 0.0019 | 0.0019 | 0.001 | 0.0016 | - | **0.002** | **0.003** |
| OI | 0.004 | 0.0034 | 0.003 | 0.0043 | 0.0022 | - | **0.024** |
| GI | 0.0021 | 0.0027 | 0.0035 | 0.0019 | 0.0024 | 0.0028 | - |

^a^ The *C*_kf_ values between offshore island soil communities are listed in the lower part of the matrix. Values in bold are *P* < 0.05.

**Table S2.** Correlations of bacterial community composition and diversity by soil properties for (a) granite islands, (b) andesite islands and (c) all islands by using the Mantel test^a^.

**(a)**

|  | Composition | Diversity |
| --- | --- | --- |
| pH | 0.10 | **0.23** |
| Organic carbon | 0.13 | 0.05 |
| Total nitrogen | **0.23** | 0.11 |
| Microbial biomass C | **0.20** | -0.06 |
| Microbial biomass N | **0.22** | 0.03 |
| Microbial biomass P | -0.03 | 0.09 |

**(b)**

|  | Composition | Diversity |
| --- | --- | --- |
| pH | 0.05 | 0.02 |
| Organic carbon | **0.51** | **0.77** |
| Total nitrogen | 0.03 | **0.45** |
| Microbial biomass C | **0.72** | **0.80** |
| Microbial biomass N | **0.47** | **0.72** |
| Microbial biomass P | **0.75** | **0.88** |

**(c)**

|  | Composition | Diversity |
| --- | --- | --- |
| pH | **0.63** | **0.36** |
| Organic carbon | **0.50** | **0.26** |
| Total nitrogen | **0.49** | **0.26** |
| Microbial biomass C | **0.76** | **0.38** |
| Microbial biomass N | **0.76** | **0.38** |
| Microbial biomass P | **0.37** | **0.29** |

^a^ Values are Spearman rank correlation coefficients between bacterial composition, diversity and soil properties. Values in bold are *P* ≤ 0.05.

**Figure S1.** Bacterial (a) Chao 1 estimator and (b) rarefaction curves of offshore soil communities. Operational taxonomic units (OTUs) were calculated at the 3% evolutionary distance. Site abbreviations are in Table 1.

**(a)**


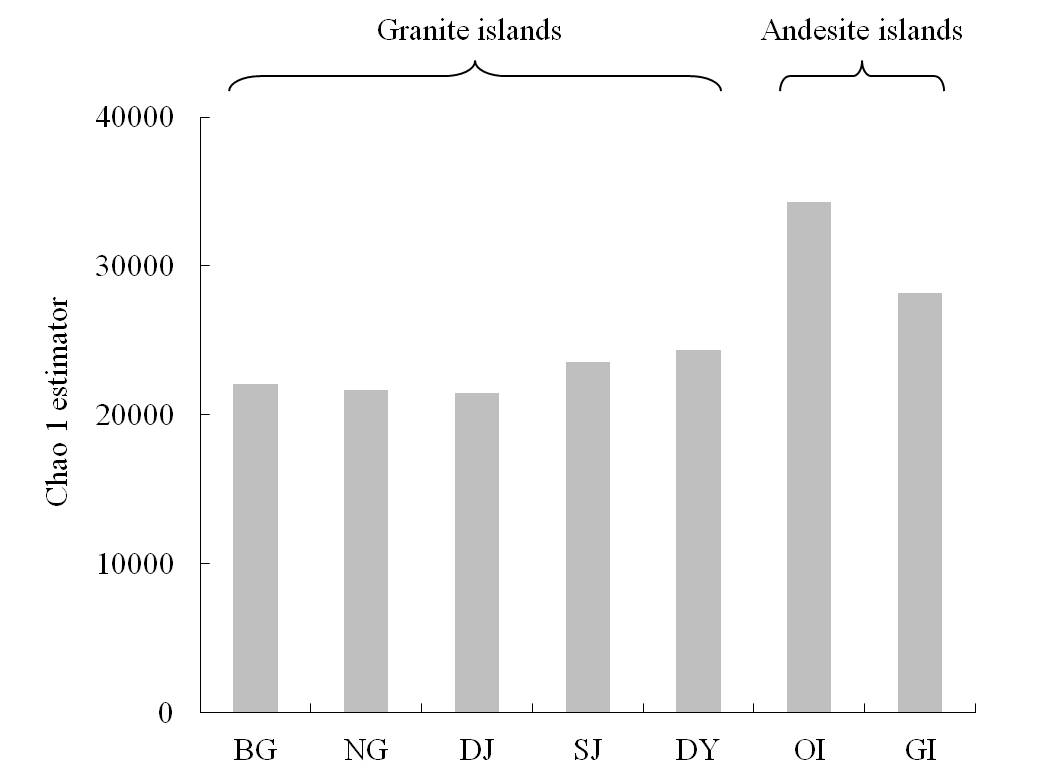


**(b)**


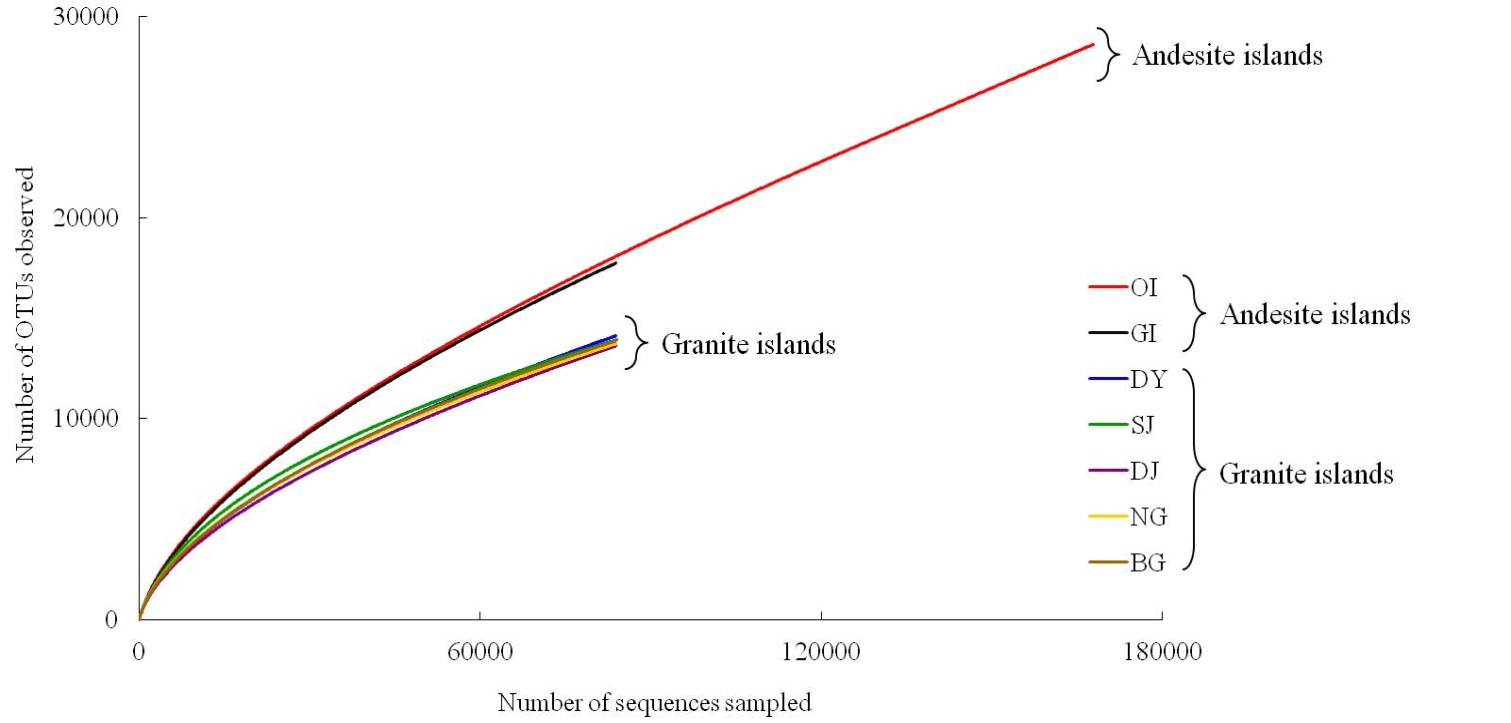


**Figure S2.** Redundancy analysis with all replicate sequences of each site among offshore soil bacterial communities. Site abbreviations are in Table 1. Abbreviations are in Figure 5.


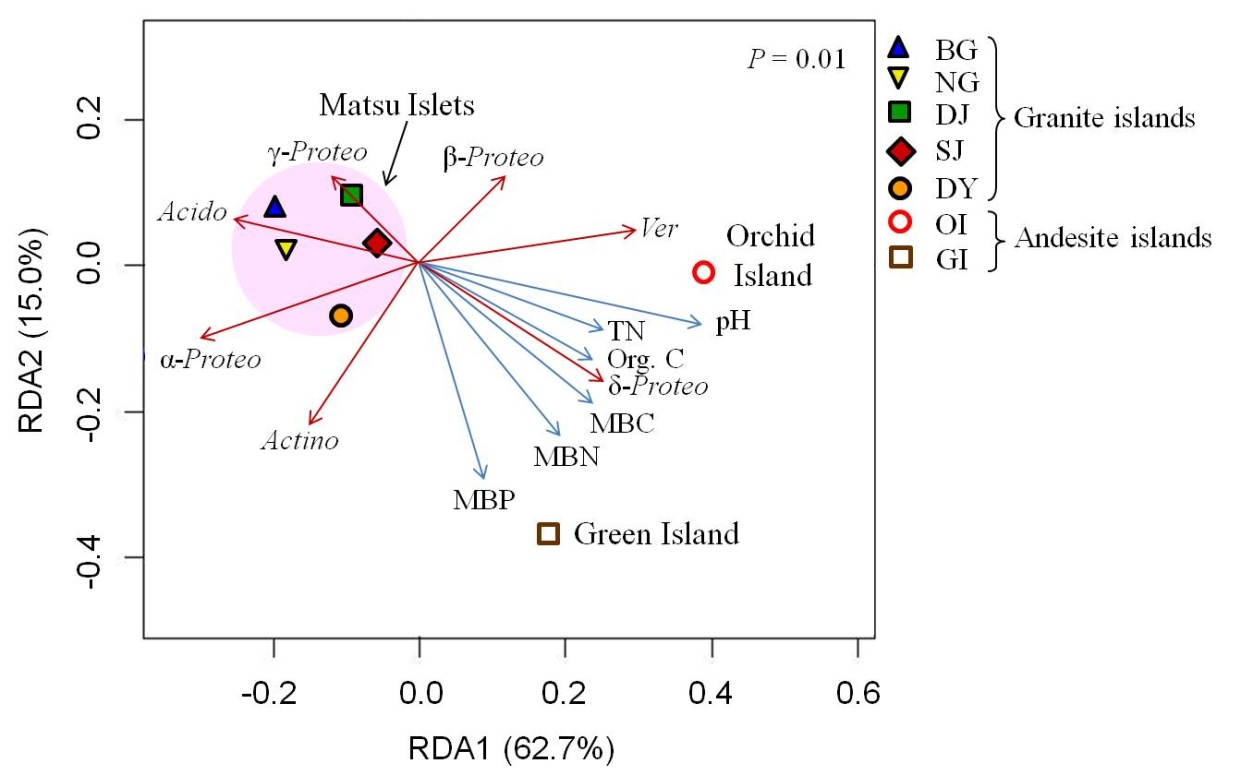

Supplement: Supplementary file 1 — Table S1, S2, and Figure S1, S2 [file 41598_2019_41170_MOESM1_ESM.docx]
